# Interictal EEG changes in patients with seizure disorder: experience in Bangladesh

**DOI:** 10.1186/2193-1801-2-27

**Published:** 2013-01-29

**Authors:** Rajib Nayan Chowdhury, ATM Hasibul Hasan, Kazi Mohibur Rahman, Badrul Alam Mondol, Sudip Ranjan Deb, Quazi Deen Mohammad

**Affiliations:** Dhaka Medical College Hospital, Dhaka, Bangladesh

**Keywords:** Interictal EEG

## Abstract

The objective of this study was to determine the changes and sensitivity of electro encephalogram during interictal period and to evaluate the finding in the clinically suspected seizure events in a tertiary care hospital of Bangladesh. This cross sectional study was carried out in the Electrophysiology Laboratory of Dhaka Medical College Hospital from July 2010 to July 2011, which included 767 patients. EEG was obtained through scalp electrodes following international 10/20 system. Patient and their attendants were interviewed using a semi structured questionnaire. The EEG findings and clinical seizure events were then compared. Among the 767 epilepsy patients most were children (39.9% less than 10 years old) and young adult (33.2% in 11–20 years age group). Female patients predominantly had seizure than male (57% and 43% respectively). The overall sensitivity of EEG in yielding abnormal interictal epileptiform discharges was 62.7%. About 48.5% of them were diagnosed as localization related epilepsy and 11.7% were generalized epilepsy. Morphology showed spike and wave in 74% and sharp and wave in 11% tracings. Only 2% had slow waves. The presence of an interictal spike/sharp wave helps to confirm a clinical diagnosis of epilepsy, aids in defining the epilepsy syndrome, provides information that assists in planning drug management.

## Introduction

Though Gibbs and his colleagues (Gibbs et al. 1935) discovered the pattern of epileptic discharges in 1935, the first description of an epileptic crisis dates back to 3000 b.c. Since the introduction, electroencephalogram (EEG) has been used to diagnose and manage epilepsy. A seizure is any clinical event caused by abnormal electrical discharge in the brain, whilst epilepsy is tendency to have recurrent seizure (Allen et al. 2010). In other words the term ‘Epilepsy’ refers to recurrent and unprovoked seizures. There is wide variation of incidence of epilepsy worldwide due to variation in classification system of epilepsy and methodology adopted in different studies (Yacubian 2000). The life time incidence of epilepsy varies from 2% to 5% (WHO report on Epilepsy in South East Asia). With the incidence of 2–10 per thousand for south East Asian countries, it is estimated that there are 1.5-2 million people suffering from epilepsy in Bangladesh (WHO report on Epilepsy in South East Asia). Marino et al (1986) showed that the incidence is highest at both extreme of ages, especially in neonatal period and after 6^th^ decade. Even with the tremendous advances in neurodiagnostic procedures, the role of EEG is not abolished. The interictal spike/sharp remains the hallmark of epilepsy, by demonstrating the cortical hyperexcitability and hypersynchrony, which may persists in the “normal” interictal state. The aim of our study was to determine the changes and sensitivity of interictal EEG in demonstrating the pathological abnormality and evaluate the presence of paroxysm of epileptiform discharges with clinically suspected seizure events. Other technologies currently used for epileptic focus lateralization, such as magneto electroencephalography (MEG) and functional magnetic resonance imaging (*f*MRI) also depend largely on analysis of interictal spikes (Sakamoto et al. 2003; Al-Asmi et al. 2003).

## Material and methods

This is a cross sectional study carried out in Electrophysiology Laboratory in Neurology Department of Dhaka Medical College Hospital (DMCH) from July 2010 to July 2011. Our study population included 767 epilepsy patients. In the Lab room meticulous history and proper physical examination was done by neurologists. All the information was kept in record files. We selected only the cases with convincing history of seizure events, which were sent for EEG. They were advised to wash hair with shampoo and not to sleep overnight, so that patient can sleep on EEG table in the following morning. EEG was done within one month of seizure event. Each recording of EEG were obtained through digital equipments with minimal duration of 20–30 minutes and electrode positioned on scalp according to international 10_˜_20 system. Recording was done in both awake and sleep state, except those who didn’t sleep, only awakened state recording was taken. Provocative stimuli like hyperventilation, photic stimulation were given for three minutes each. For standardization, the background activity was classified as normal (organized and symmetrical) or abnormal (disorganized and/or asymmetrical). The EEG was interpretated by consultant neurologist, trained and experienced in electro-physiologic studies. The EEG was examined for specific epileptiform abnormality, the interictal spike or sharp wave. The abnormal electroencephalographic activity was also classified as generalized or focal. The presence and topography of bursts of slow waves and epileptiform paroxysms were evaluated. The latter were classified as spike-wave, sharp-wave and polyspike. EEG was done within one month of last seizure and mostly after a single event. Though a total of 783 patients were initially interviewed, 16 patients with marked artifacts on EEG were excluded from this study.

## Results

**Figure 1:**
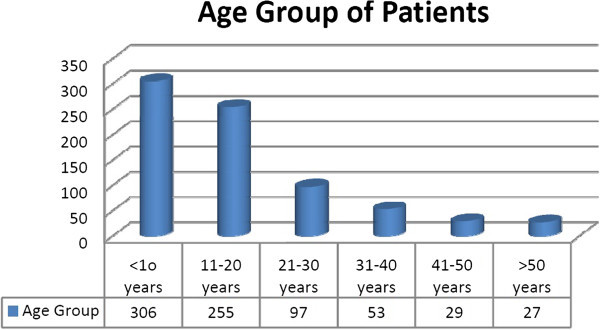
**Age group of patients.**

**Figure 2:**
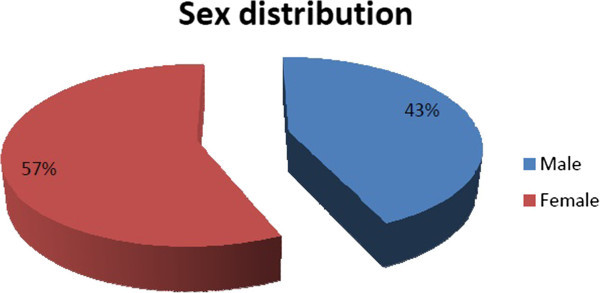
**Sex distribution epilepsy patients.**

In our study, the patient’s age ranged from birth to 64 years. The mean age at presentation to the EEG room was 9±2.36 years i.e. most of the patients were less than 10 years old (39.9%). The next common age group at seizure presentation was 11–20 years (33.2%). Only 27 patients (3.5%) were older than 50 years (Figure 1). Fifty seven percent of the patients were female and 43% were male (Figure 2). Out of 767 EEGs done in clinically diagnosed seizure patients, 294 (38.3%) were normal and 473 (62.7%) revealed abnormal. So the sensitivity of EEG in detecting electroencephalographic alteration in clinically suspected epileptic patients was 62.7% (about 69.6% below 20 years and 43.7% in those above 20 years). Among them 48.5% was diagnosed as localization related epilepsy (LRE) and 11.7% had generalized (GE) epileptiform discharge (Table 1). Among the patients with LRE, most common focus was temporal lobe (15.4%) (Table 1). Regarding the morphology of epileptiform discharge, 74% (341) had spike and wave, 11% sharp and wave, 6% poly spikes and only 2% had slow waves (Table 2). Disorganized and or asymmetric background was recorded in 9% patients who had diagnosis of encephalopathy (12,2.6%), mental retardation (19, 4%) and cerebral palsy (11, 2.4%).

**Table 1:** **Inter-ictal EEG of patients (N=767)**

EEG Findings	n	%
Normal	294	38.3
Abnormal	473	62.7
Localization Related Epilepsy	372	48.5
Frontal	51	6.6
Temporal	118	15.4
Parietal	53	6.9
Occipital	19	2.4
Central	74	9.6
Others	57	7.4
Generalized Epilepsy	90	11.7
Others	11	1.5

Shows that out of 767 patients 62.7% (n-473) had abnormal EEG findings. Among these 48.5% (n-372) were LRE.

**Table 2:** **Morphology of abnormal epileptiform discharge (N=473)**

EEG Findings	n	%
Abnormal background activity	42	9
Spike and wave	341	74
Sharp and wave	51	11
Poly spikes	29	6
Slow waves	10	2

Shows that Spike and wave were the predominant epileptiform activity comprising 74% (n-341).

## Discussion

Epileptiform activity is quite specific for a diagnosis of epilepsy as the cause of transient loss of consciousness or other paroxysmal event which is clinically like to be epilepsy. In epileptic patients the EEG is examined for a specific epileptiform activity, the interictal spike or sharp wave. The spike or sharp wave represents the summated excitatory and inhibitory postsynaptic potentials associated with hyper-synchronous neuronal firing with paroxysmal depolarization shift and aftergoing hyperpolarization. This differing appearance, though not significant, reflects the rapidity of neuronal synchronization and the way in which the discharge spreads through the cortex. These discharges may either be focal or generalized. Since the beginning of use of EEG, it always has been a matter of query that how often the EEG does detect an interictal spike or sharp wave in an epileptic patient. Most of the patients in our study were children. This is probably due to the fact that parents are often frightened to observe a seizure event in their kid which led them to seek expert advice reasonably early. On the other hand adult patients are often reluctant to consult neurologists and often they prefer imaging first for clue to diagnosis of seizure etiology. Like our study, most of the reports regarding sensitivity of EEG are retrospective evaluation of data base from tertiary care centers. Several published studies on adult epilepsies showed that the chance of detecting interictal epileptiform discharges (IEDs) from the first EEG varies between 29% and 55% (Marsan & Zivin 1970; Goodin et al. 1990; Salinsky et al. 1987) at outdoor monitoring of patients. Repeated EEG ultimately demonstrated the IEDs in 80%-90% of the patients (Marsan & Zivin 1970; Salinsky et al. 1987). In video EEG monitoring also yields similar results. Walczak et al (1993) showed that long term video EEG monitoring can detect IEDs in up to 81% of the patients. Adoption of several methods can increase the chance of detecting IEDs. Sleep effectively improves detection of both generalized and focal IEDs (1975; Niedermeyer & Rocca 1972; Sato et al. Angeleri 1973). It is also likely to occur in an EEG if recorded early after a seizure event (Niedermeyer & Rocca 1972). Hyperventilation and photic stimulation also induces IEDs in many patients, especially in generalized seizures (Sato et al. 1983; Miley & Forster 1977; Wolf & Goosses 1986). In healthy adult with no history of declared seizure, the incidence of epileptiform discharge is only 0.5%. This also confers high sensitivity of EEG in detecting abnormal discharges (Gregory et al. 1993). Similar to other published reports, our study yielded abnormal EEG in most of the cases (62.7%). Kershman et al (1951) reported in a series of 2648 patients with unquestionable diagnosis of seizure that 46.5% had focal changes while 15% had diffuse generalized abnormality in their scalp recordings of EEG. Our sample did not differ much in distribution of focal and generalized discharge (48.5% and 11.7% respectively). In contrast to our finding, Dantas et al (2005) showed a lower sensitivity (43.6%) of EEG, which probably accounts for the difference in age group of sample population. Betting et al. (2006) in his retrospective analysis of 493 EEG tracing from 180 generalized epilepsy patients showed that 33% had generalized spike or polyspikes, while 22% had focal discharges in children. Morphologic analysis of EEG tracing in our study revealed a marked preponderance of spike wave paroxysms which comply with higher frequency of focal discharges.

We had some limitations. Firstly, there is chance of sampling bias. Moreover, the chance of inter observer biasness was minimized by following same principle in recording and typing the EEG abnormality.

## Conclusion

The EEG is an important tool for diagnosing an epilepsy syndrome, knowledge of which aids in planning treatment and determining prognosis. Bangladesh being a third world country, has limited investigation facilities for epilepsy patients. Dhaka Medical College is running EEG lab. for a long time. And in Government level it is the only tertiary care hospital providing this facility. People from all sections and all corners of the country get the service from this hospital. So, the data presented here, gives us the actual scenario of EEG changes in patients with seizure disorder, for the first time. The presence of focal IEDs suggests the diagnosis of a localization related epilepsy, the character and location of which offer clues to both the etiology of the epilepsy syndrome and the location of the epileptogenic region. Finding of a generalized IED suggests the diagnosis of one of the generalized epilepsy syndromes. Thus the interictal EEG serves several purposes especially aids in detecting whether the epilepsy is present or not, helps in classifying the seizure disorder and also defining the epilepsy syndromes. With proper equipment and duration of recording through scalp electrodes it can detect abnormal cerebral neuronal discharges in two third cases of clinical seizure events. The finding of our study further consolidated the concept that EEG still remains the key investigation in clinically suspected epileptic patients and has a high level of sensitivity when performed in conditions properly indicated.

### Ethics

The study protocol was approved by institutional ethical committee of Dhaka Medical College Hospital.

## Data sharing

There is no other unpublished data to share.

## References

[CR1_106] Al-Asmi A Bennar CG Gross DW fMRI activation in continuous and spike-triggered EEG- fMRI studies of epilepsy spikes Epilepsia 2003 44 1328 39 10.1046/j.1528-1157.2003.01003.x 14510827

[CR2_106] Allen CMC Lueck CJ Dennis M Colledge NR Walker BR Ralston SH Neurologic disease Davidson’s Principles and Practice of Medicine 2010 21 1172

[CR3_106] Angeleri F Levin P Koella W Partial epilepsies and nocturnal sleep Sleep 1975 Basel Karger 196 203

[CR4_106] Betting LE Mory SB Lopes-Cendes I EEG features in idiopathic generalized epilepsy: clues to diagnosis Epilepsia 2006 47 3 523 8 10.1111/j.1528-1167.2006.00462.x 16529616

[CR5_106] Dantas FG Medeiros JLA Nogueira BNF Papel do EEG em casos de suspeita ou diagnóstico de epilepsia J Epilepsy Clin Neurophysiol 2005 11 2 77 8 10.1590/S1676-26492005000200002

[CR6_106] Gibbs FA Davis H Lennox WB The electroencephalogram in epilepsy and in conditions of impaired consciousness Arch Neurol Psychiatry 1935 34 1133 48 10.1001/archneurpsyc.1935.02250240002001

[CR7_106] Goodin DS Aminoff MJ Laxer KD Detection of epileptiform activity by different noninvasive EEG methods in complex partial epilepsy Ann Neurol 1990 27 330 4 10.1002/ana.410270317 2327741

[CR8_106] Gregory RP Oates T Merry RTG EEG epileptiform abnormalities in candidates for aircrew training Electroencephalogr Clin Neurophysiol 1993 86 75 7 10.1016/0013-4694(93)90069-8 7678394

[CR9_106] Kershman J Vasques J Golstein S The incidence of focal and non-focal EEG abnormalities in clinical epilepsy Electro encephal Clin Physiol 1951 3 15 24 10.1016/0013-4694(51)90050-8 14822918

[CR10_106] Marino R Jr Cukiert A Pinho E Aspectos epidemiológicos da epilepsia em São Paulo: um estudo de prevalência Arq Neuro Psiquiatr 1986 44 3 243 54 3496069

[CR11_106] Marsan CA Zivin LS Factors related to the occurrence of typical paroxysmal abnormalities in the EEG records of epileptic patients Epilepsia 1970 11 361 81 10.1111/j.1528-1157.1970.tb03903.x 5278205

[CR12_106] Miley CE Forster FM Activation of partial complex seizures by hyperventilation Arch Neurol 1977 34 371 3 10.1001/archneur.1977.00500180065014 871260

[CR13_106] Niedermeyer E Rocca U The diagnostic significance of sleep electroencephalograms in temporal lobe epilepsy. A comparison of scalp and depth tracings Eur Neurol 1972 7 119 29 10.1159/000114418 5019152

[CR14_106] Sakamoto S Takami T Hara M Interictal pattern of cerebral glucose metabolism, perfusion and magnetic field in mesial temporal lobe epilepsy Epilepsia 2003 44 1196 1206 10.1046/j.1528-1157.2003.08603.x 12919392

[CR15_106] Salinsky M Kanter R Dasheiff RM Effectiveness of multiple EEGs in supporting the diagnosis of epilepsy: an operational curve Epilepsia 1987 28 331 4 10.1111/j.1528-1157.1987.tb03652.x 3622408

[CR16_106] Sato S Dreifuss FE Penry JK The effect of sleep on spike-wave discharges in absence seizures Neurology 1973 23 1335 45 10.1212/WNL.23.12.1335 4357115

[CR17_106] Sato S Dreifuss FE Penry JK Long-term follow-up of absence seizures Neurology 1983 33 1590 5 10.1212/WNL.33.12.1590 6417557

[CR18_106] Walczak TS Scheuer ML Resor S Prevalence and features of epilepsy without interictal epileptiform discharges Neurology 1993 43 suppl 287 8

[CR19_106] WHO report on Epilepsy in South East Asia: *Some facts and figures in Epilepsy*. Avaialable at: http://www.searo.who.int/LinkFiles/Information_and_Documents_facts

[CR20_106] Wolf P Goosses R Relation of photosensitivity to epileptic syndromes J Neurol Neurosurg Psychiatry 1986 49 1386 91 10.1136/jnnp.49.12.1386 3806115 PMC1029123

[CR21_106] Yacubian EMT Yacubian EMT Epilepsia: o conceito atual Epilepsia da Antiguidade ao Segundo Milênio 2000 São Paulo Lemos 82 8

